# PCBs Exert an Estrogenic Effect through Repression of the *Wnt7a* Signaling Pathway in the Female Reproductive Tract

**DOI:** 10.1289/ehp.8748

**Published:** 2006-02-02

**Authors:** Risheng Ma, David A. Sassoon

**Affiliations:** Brookdale Department of Molecular, Cell, and Developmental Biology, Mount Sinai Medical School, New York, New York, USA

**Keywords:** Aroclor 1254, DES, diethylstilbestrol, endocrine disruptor, female reproductive tract, PCBs, polychlorinated biphenyls, *Wnt7a*

## Abstract

Polychlorinated biphenyls (PCBs) have been proposed to have a weak estrogenic activity and therefore pose a risk as potential environmental endocrine disruptors to the perinatal development of the female reproductive tract. Perinatal exposure to high concentrations of the potent synthetic estrogen diethylstilbestrol (DES) induces abnormal development of the female reproductive tract via a mechanism that acts through the down-regulation of *Wnt7a* (wingless-type MMTV integration site family, member 7A). To test the hypothesis that PCBs act as weak estrogens, we injected neonatal mice with a commercial PCB mixture (Aroclor 1254) or with low levels of DES and measured effects of exposure on *Wnt7a* expression and uterine morphology. We report here that neonatal PCB or low-level DES exposure resulted in the down-regulation of *Wnt7a* expression. In addition, both PCB and low-level DES exposure induced changes in the uterine myometrium and gland formation. These data reveal that weak estrogens such as the PCBs act through a *Wnt7a*-dependent pathway and suggest that *Wnt7a* regulation is a sensitive biomarker for testing weak estrogenic candidate compounds. The morphologic changes that were elicited by PCBs and DES were different immediately after exposure, suggesting that *Wnt7a*-independent pathways are also activated by one or both of these compounds. Although *Wnt7a* down-regulation is transient after estrogenic exposure, subsequent morphologic changes became more pronounced during postnatal and adult life, suggesting that the female reproductive tract is permanently reprogrammed after exposure even to weak estrogenic compounds. In addition, *Wnt7a* heterozygous mice were more sensitive to PCB exposure, revealing an important genetic predisposition to risks of environmental endocrine disruptors.

Polychlorinated biphenyls (PCBs) are a large group of aromatic chlorinated hydrocarbons that were first introduced in the late 1920s and commercially produced as Aroclor mixtures in the United States (Battershill 1994; Lind et al. 1999; Safe 1997; Safe and Zacharewski 1997; Shekhar et al. 1997b; Shipp et al. 1998). These compounds were used widely in industry for a wide variety of purposes ranging from plastic resins to carbonless copy paper. Despite their utility, PCBs are linked to environmental and human health risks (Safe 1997; Safe and Zacharewski 1997; Svendsgaard et al. 1997; Swanson et al. 1995; Tilson and Kodavanti 1997) that have resulted in the subsequent banning or highly restricted use of PCBs in many countries, including the United States. However, it is estimated that > 1 million tons of PCBs have been produced, and > 70% of the PCBs made are still in use (Adami et al. 1995; Ahlborg et al. 1995; Battershill 1994; Brouwer et al. 1999; Colborn and Smolen 1996; Davidson 1998; Safe 1997). Because of the high stability of PCBs, they persist in the environment and have been detected throughout the food chain (Gladen et al. 1999; Little et al. 1999; Longnecker et al. 1999; Rogan et al. 1999). Of direct concern to public health is the reported accumulation of PCBs in human tissues, breast milk, and reproductive organs (Adami et al. 1995; Ahlborg et al. 1995; Baker 2001; Battershill 1994; Eddy et al. 1996; Fielden et al. 1997; Hany et al. 1999; Nesaretnam et al. 1996; Nicolopoulou-Stamati and Pitsos 2001; Ogawa et al. 1999; Safe 1997; Safe and Zacharewski 1997).

The toxic and biochemical effects of commercial PCB mixtures have been extensively investigated in a variety of laboratory animals and wildlife species. PCBs have been demonstrated to alter endocrine, immune, and nervous system functions and cause adverse effects on reproductive and development of animals, including humans. After maternal exposure to PCBs, decreased embryonic growth, delayed implantation, and increased abortion rates have been observed (Kholkute et al. 1994; McNulty 1985; Seiler et al. 1994). PCBs, like the structurally related DDT (dichloro-diphenyltrichloroethane) family of pesticides, appear to have specific effects upon sexual development and reproductive function in animals and humans. Although studies on PCB mixtures have been extensive over the last several decades, a mechanistic understanding of how PCBs alter reproductive function and development has been elusive. A confounding aspect of PCBs research is the fact that different animal models give rise to different and often conflicting outcomes.

PCBs have been proposed to act through a variety of cellular pathways. Of particular interest to our studies is the potential estrogenic activity of these compounds that have been demonstrated *in vitro* and *in vivo* (Arcaro et al. 1999; Hany et al. 1999; Shekhar et al. 1997a). These studies reveal that the estrogenic properties of PCBs are weak (Lind et al. 1999), whereas some PCB mixtures in other systems exhibit antiestrogenic activity (Ramamoorthy et al. 1997). However, it is possible that even weak estrogenic exposure during early development may have a profound impact upon post-natal development. This phenomenon, often referred to as endocrine disruption, was first realized with the appearance of uterine and vaginal/cervical cancers in young women whose mothers took the synthetic estrogen diethylstilbestrol (DES) during pregnancy (Hatch et al. 1998; Kitajewski and Sassoon 2000; Mericskay et al. 2005; Palmer et al. 2002; Saunders 1988). In this case, pathologies did not appear until 2–3 decades after initial exposure, leading to the eventual withdrawal of DES from prenatal care in the 1970s, almost 40 years after initial clinical use (Hatch et al. 1998; Kitajewski and Sassoon 2000; Mericskay et al. 2005; Palmer et al. 2002; Saunders 1988). DES is a very potent estrogenic compound that contributed to the comparatively rapid elucidation of the endocrine disruptor risks posed by its use. By comparison, the obstacles in identifying weak endocrine disruptors present in the environment are considerable. It is likely that events that occur during fetal and/or perinatal life that have a negative impact on the adult alter normal embryonic and fetal developmental programs. Thus, a mechanistic understanding of fetal endocrine disruption will necessitate an understanding of the fundamental mechanisms underlying normal development of target organ systems.

We have demonstrated previously that *WNT* genes direct the proper cytodifferentiation and overall development of the Müllerian-derived female reproductive tract. Analyses of two *WNT* mouse mutants (*Wnt7a* and *Wnt5a*; wingless-type MMTV integration site family, members 7A and 5A, respectively) have demonstrated that *WNT* gene activity is crucial for proper development and subsequent adult function (Carta and Sassoon 2004; Mericskay et al. 2004, 2005; Miller and Sassoon 1998; Miller et al. 1998a). We have demonstrated that *Wnt7a* is down-regulated by DES exposure during a critical perinatal period of development and that this transient down-regulation of *Wnt7a* is sufficient to recapitulate the effects of a complete loss of function of *Wnt7a* in the female reproductive tract. DES-exposed wild-type and *Wnt7a* mutant mice show aberrant morphology by 1–2 months after birth and precancerous and bona fide tumors by 9–18 months after birth (Miller et al. 1998a). Although these studies provide strong genetic evidence that *Wnt7a* is a primary target of DES exposure, it is important to note that these studies were performed using very high DES concentrations [~ 300 μg/g body weight (bw)], similar to those once used for pregnant women (Carta and Sassoon 2004; Mericskay et al. 2004; Miller et al. 1998a). Nonetheless, our data suggest that the negative regulation of *Wnt7a* expression is an early and key event that signifies pathologic risks that appear much later in life. We thus set out to determine if *Wnt7a* is deregulated in response to potential weak estrogenic compounds such as PCBs, by comparing the molecular and cellular responses to both PCBs and very low concentrations of DES (10 ng/g bw). We demonstrate here that Aroclor 1254 or low levels of DES are capable of down-regulating *Wnt7a* expression in the neonatal female reproductive tract similar to that observed after high concentrations of DES. Unexpectedly, we found that Aroclor 1254 led to a different phenotypic outcome compared with DES exposure during early postnatal development; however, by postnatal day (PND) 30, DES and Aroclor 1254 exposure resulted in a similar altered phenotype in the uterus, including changes in the myometrium and glandular content. We also found that mice carrying a homozygous null mutation for *Wnt7a* were insensitive to phenotypical changes caused by either low-level DES exposure or exposure to Aroclor 1254, consistent with a central role for *Wnt7a* in estrogenic endocrine disruptor events. *Wnt7a* heterozygous mice showed an increased sensitivity to Aroclor 1254, demonstrating the potential for genetic predisposition to endocrine disruption.

## Materials and Methods

### Chemicals

DES (lot 98H0715; Sigma-Aldrich, St. Louis, MO) was dissolved in a 10% ethanol saline solution (0.85% NaCl) at a concentration of 0.2 μg/mL. The PCB mixture Aroclor 1254 (lot 124–191; AccuStandard, New Haven, CT) was dissolved in a 10% ethanol saline solution at a concentration of 12.5 mg/mL.

### Animals and treatment

*Wnt7a* heterozygous mice were originally obtained from B. Parr and A. McMahon (Parr and McMahon 1995, 1998) and maintained on an SV129 background. All animals were maintained in plastic cages and housed in a temperature-controlled room (21–22°C) under a 12-hr light/12 hr dark schedule. All mice used for this study were generated from heterozygous crossings. Mice were provided fresh reverse-osmosis/deionized water and NIH-31 lab chow (National Institutes of Health, Bethesda, MD) *ad libitum*. Pups were injected subcutaneously with DES (10 ng/g bw/day) or Aroclor 1254 (500 μg/g bw/day) from postnatal day 1 (PND1) to PND5. Controls were similarly treated with the same volume of vehicle. Mice were killed by cervical dislocation. All procedures for handling of mice, housing, and maintenance were performed according to approved institutional guidelines that take into account the humane treatment and all required safeguards to minimize suffering.

### Tissue processing and cell counts

Tissues were removed and processed for histology as previously described (Carta and Sassoon 2004; Mericskay et al. 2004; Miller, et al. 1998b; Sassoon and Rosenthal 1993). Briefly, reproductive tracts were dissected in cold phosphate-buffered saline (PBS) and fixed overnight in 4% PBS-buffered paraformaldehyde. After dehydration, tissues were embedded in paraffin, sectioned at 6 μm, and stained with hematoxylin and eosin (H&E). Comparative photographs of wild-type and mutant saline-treated, DES-treated, and Aroclor 1254–treated mice were taken at the same magnification. Epithelial cell counts, gland number, and the thickness of the smooth muscle layer were obtained from at least three sections per individual and at least three individuals per group. Specifically, total luminal epithelium cell number was counted per single whole-uteri cross section; we counted three sections per individual and three individuals per group were counted. Gland number was counted per single whole-uteri cross section; three sections per individual and three individuals per group were counted. Completely formed glands were counted as one gland, and invaginating luminal epithelium in the process of forming glands was counted as a half gland. We measured the thickness of smooth muscle from sections that were stained for smooth muscle actin obtained from three different uteri cross sections. Three sections per individual and three individuals per group were measured. In all cases, sections were obtained at various rostral–caudal regions to avoid differences due to the specific location within the uterine horn. Statistical evaluation was made using the unpaired Student *t*-test. Statistical significance was assigned at *p* < 0.05. All *p*-values that are significant or that indicate a trend are shown as either *p* < 0.05 or *p* < 0.01.

### In situ *hybridization*

*In situ* hybridization was performed as described previously (Carta and Sassoon 2004; Mericskay et al. 2004; Miller, et al. 1998b; Sassoon and Rosenthal 1993). Antisense ^35^S-labeled riboprobes were generated for *Wnt7a* (Miller and Sassoon 1998; Miller et al. 1998a; Sassoon and Rosenthal 1993). Black-and-white dark-field images were converted to reverse-red grains and superimposed upon standard light microscope images using Adobe Photoshop (Adobe Systems Inc., San Jose, CA), allowing for easy identification of labeled structures.

### Immunohistochemistry

Tissue was processed for paraffin histology as described above. After sections were deparaffinized and rehydrated, they were blocked for 1 hr in 10% goat serum, 0.1% Triton X-100, 1% bovine serum albumin, and 0.2% gelatin. They were then incubated for 1 hr with a monoclonal anti-smooth muscle actin at a final dilution of 1:50 in blocking buffer. Sections were washed and incubated in horseradish peroxidase (HRP)-conjugated anti-mouse IgG secondary antibodies (Jackson ImmunoResearch Laboratories, Inc., West Grove, PA) at a final dilution of 1:500 in blocking buffer for 1 hr at room temperature. Bound mouse antibodies were detected using streptavidin-HRP (Zymed Laboratories, San Francisco, CA) with amino-ethyl carbazole as substrate.

### *Quantitative reverse-transcriptase poly-merase chain reaction (RT-PCR) for* Wnt7a

Total RNA was isolated using TRIzol reagent (Invitrogen, Carlsbad, CA) according to the manufacture’s protocol. Concentrations of the final preparations were calculated from an A260 reading (Beckman DU-7 spectrophotometer; Beckman Coulter Inc., Fullerton, CA, USA) and an aliquot analyzed by gel electrophoresis to ensure integrity. Reverse transcriptase generation of cDNA was carried out in a reaction mix consisting of 1.5 μg random primer (Invitrogen), 0.2 mM (d)NTPs (Omniscript kit; Qiagen, Valencia, CA, USA), 40 U RNase inhibitor (Omniscript kit, Qiagen), 4 U Omniscript RT (Omniscript kit, Qiagen), and 2 μg total RNA in a volume of 20 μL. Quantitative RT-PCR for *Wnt7a* was simultaneously performed on this cDNA preparation from total RNA from tissues of three different animals for each treatment group using the LightCycler-based Sybr Green I detection system (Roche Molecular Systems Inc., Branchburg, NJ, USA). Negative controls without cDNA were used to assess specificity. A stable housekeeping gene, glyceraldehyde-3-phosphate dehydrogenase (GAPDH), was used to control for input RNA. The reactions were prepared for each cDNA sample as follows: 20 μL reaction consisting of 10 μL Sybr Green optimized buffer (Qiagen), 20 pmol of each of GAPDH primer or *Wnt7a* primer, and 1 μL cDNA. Thermal cycling conditions were as follows: initial 94°C/15 min, 94°C/15 sec, 55°C/25 sec, 72°C/10 sec, and 40°C/30 sec. The process was carried out in duplicate for each cDNA preparation. The amount of transcript was determined based on the plot of fluorescence versus cycle number. After adjusting with the GAPDH control, the differences in cycle crossing points were calculated for each group for a specific time point. For a theoretical efficiency of 100%, the fold difference was calculated by 2 to the power of the cycle point difference. Results are normalized for GAPDH, and data are shown as mean ± SD from three independent experiments run in duplicate.

## Results

### *Aroclor 1254 exposure results in down-regulation of* Wnt7a

**Figure 1:**
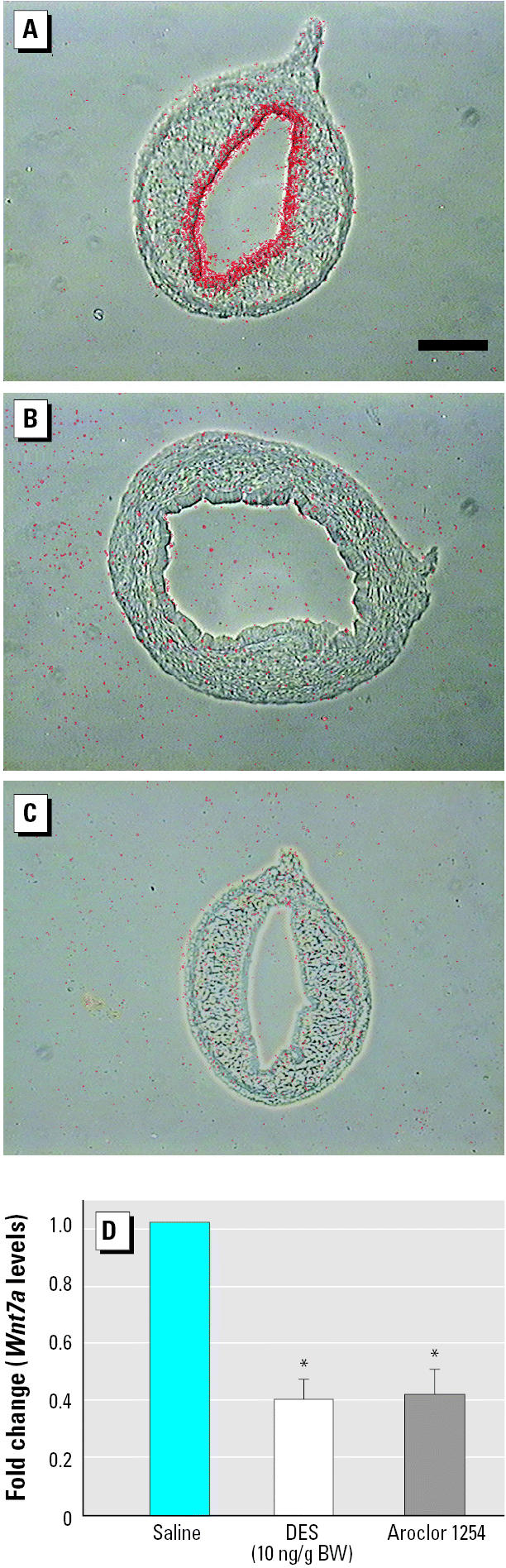
Cross sections from wild-type uterine horn at PND6 after injection of saline (*A*), DES (*B*), or Aroclor 1254 (*C*) from PND1 to PND5 hybridized with probe corresponding to *Wnt7a. Wnt7a* transcripts were detected throughout the luminal uterine epithelium of saline-treated mice (*A*). DES or Aroclor 1254 exposure resulted in down-regulation of *Wnt7a* expression (*B* and *C*, respectively). Photomicrographs are composites of phase and dark field (red) for direct comparison of *in situ* signal on tissue sections. Bar = 100 μm. (*D*) Real-time RT-PCR confirming that *Wnt7a* expression was down-regulated 24 hr after the final DES or Aroclor 1254 injection (PND6). Error bars indicate SD calculated from *n* ≥ 3 using the unpaired Student *t*-test. *p < 0.05 compared with saline.

Perinatal exposure to high levels of DES (200 μg/day) resulted in a down-regulation of *Wnt7a* transcripts in the luminal epithelium of the uterus (Miller et al. 1998a). As a first step to assess the estrogenic effects of Aroclor 1254, we first tested whether low concentrations of DES were capable of repressing *Wnt7a* expression because previous studies suggested that Aroclor 1254 has only weak estrogenic activity (Brown and Lamartiniere 1995; Jansen et al. 1993, Shekhar et al. 1997a). We injected neonatal pups once a day from PND1 to PND5 with DES at a dose of 10 ng/g bw, which is similar to levels used by other laboratories to establish a baseline for weak estrogens (Newbold et al. 2001) and is > 500-fold lower than levels used in our previous studies (Carta and Sassoon 2004; Mericskay et al. 2004; Miller et al. 1998a). The effects of DES and Aroclor 1254 upon *Wnt7a* gene expression were assessed using *in situ* hybridization of uterine tissues from PND6 mice collected 1 day after the last injection. Control mice were injected with saline solution, which was used as a vehicle for both DES and Aroclor 1254. As expected, *Wnt7a* expression was detected throughout the luminal epithelium in the mouse uterus of saline-injected mice (Figure 1A). In contrast, exposure to low levels of DES was sufficient to down-regulate *Wnt7a* to levels undetectable with *in situ* hybridization (Figure 1B). In addition, exposure to Aroclor 1254 resulted in a down-regulation of *Wnt7a* transcripts (Figure 1C). Our *in situ* analyses of DES-exposed and Aroclor 1254–exposed uteri showed variable results with occasional low but detectable levels of signal in the uterine epithelium, suggesting that Aroclor 1254 exposure and the lower levels of DES used in these studies may not completely shut down *Wnt7a* transcription. To confirm this result, we used quantitative PCR and observed that *Wnt7a* transcripts were reduced by approximately 60% in response to DES or Aroclor 1254 exposure (Figure 1D). These results reveal that the uterus responds to Aroclor 1254 by down-regulating *Wnt7a*, suggesting that Aroclor 1254 causes effects through mechanisms similar to those affected by DES. Our quantitative analyses of *Wnt7a* levels using RT-PCR revealed a baseline level of *Wnt7a* transcripts after DES and/or Aroclor 1254 exposure, whereas *in situ* hybridization revealed no detectable levels of *Wnt7a* transcripts. This is likely due to both the higher sensitivity of the RT-PCR approach and the necessity to normalize our data to GAPDH. However, the RT-PCR data were obtained from entire uterine horns in which only a subset of the cells (luminal epithelium) express *Wnt7a*. These data therefore confirm our *in situ* results and reveal a similar efficacy of DES and Aroclor 1254 in down-regulating *Wnt7a*.

### *Aroclor 1254 and DES effects are dependent upon* Wnt7a *genotype: evidence for convergent and divergent pathways.*

**Figure 2:**
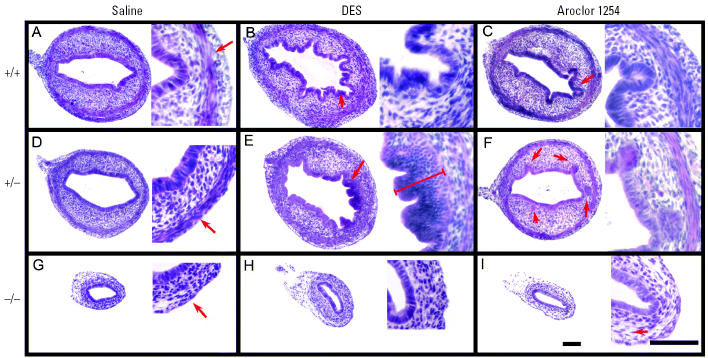
Photomicrographs (two magnifications each) of representative H&E-stained cross sections of wild-type, *Wnt7a* +/−, and *Wnt7a* −/− uteri analyzed on PND6, 24 hr after final injection of saline, DES, or Aroclor 1254. Saline-treated wild-type (*A*) and *Wnt7a* heterozygous uteri (*D*) were indistinguishable with regard to overall size, epithelial morphology, stroma, and myometrial differentiation (arrows). In contrast, the diameter of the *Wnt7a* mutant uterus (*G*) was smaller and the myometrium was not visible (arrows). Low-level DES exposure resulted in epithelial folding in wild-type uteri (*B*). In the *Wnt7a* +/− uteri (*E*), the epithelium underwent stratification. In contrast to the effects of DES, Aroclor 1254 exposure induced precocious gland formation (arrows) in both wild-type (*C*) and *Wnt7a* +/− uteri (*F*). In contrast, *Wnt7a* −/− uteri showed no consistent or overt changes in morphologies in response to DES (*H*) or Aroclor 1254 (*I*) exposure, although we noted signs of water imbibition, as demonstrated by an increase in the intercellular spaces (arrows). Bar = 100 μm.

Our results reveal that both Aroclor 1254 and low-level DES exposure provoked a down-regulation of *Wnt7a*. We had shown previously that loss of *Wnt7a* function in mice leads to the same phenotypic outcome in the uterus as does perinatal exposure to high levels of DES (Miller and Sassoon 1998; Miller et al. 1998a). We would therefore predict that exposure to either Aroclor 1254 or low levels of DES should result in a similar phenotypic outcome due to the down-regulation of *Wnt7a*. Furthermore, if down-regulation of *Wnt7a* is a primary event in response to either compound, we would predict that *Wnt7a* heterozygous mice (*Wnt7a* +/−) are more sensitive to weak estrogens compared with wild-type mice (*Wnt7a* +/+). We therefore examined uterine horns from wild-type, *Wnt7a* +/−, and *Wnt7a* −/− mice exposed to either low levels of DES or Aroclor 1254 during PND1–PND5. As shown in Figure 2, control (saline-injected) wild-type and *Wnt7a* +/− uterine horns had very similar morphologies (Figure 2A,D), whereas the control *Wnt7a* −/− uterine horns were small and atrophic in appearance (Figure 2G). By PND6, the uterine myometrium began to differentiate in the wild-type and heterozygote uterine horns, whereas no myometrium can be identified in the *Wnt7a* −/− uterus at this stage (Figure 2G). Exposure to low levels of DES elicited pronounced folding of the uterine luminal epithelium as well as a flattened apical appearance to the epithelial cells in wild-type uteri (Figure 2B). A more pronounced effect was observed in the *Wnt7a* +/− uterine epithelium, which became stratified (Figure 2E). This is reminiscent of the effects we reported in wild-type uteri exposed to high levels of DES during perinatal development (Miller et al. 1998a) suggesting that *Wnt7a* heterozygote uteri are more sensitive to low levels of estrogenic compounds. In contrast, Aroclor 1254 exposure did not induce the same morphologic outcomes as DES. Significantly less luminal folding was observed in response to Aroclor 1254 in both the wild-type and *Wnt7a* +/− samples; however, distinct uterine glands formed precociously (Figure 2C,F). We interpret these results to suggest that either the precise levels of estrogenic stimulation are critical for the morphologic response, or that both DES and Aroclor 1254 function through *Wnt7a*-dependent and -independent pathways. For both DES and Aroclor 1254, *Wnt7a* was similarly down-regulated; thus, additional uncharacterized molecular and cellular responses must exist that are distinct for each compound. Exposure to low levels of DES or Aroclor 1254 had no obvious effect upon the *Wnt7a* −/− uterus, although we noted occasional signs of water imbibition in some samples as indicated by increased intercellular spaces (Figure 2H,I).

**Figure 3:**
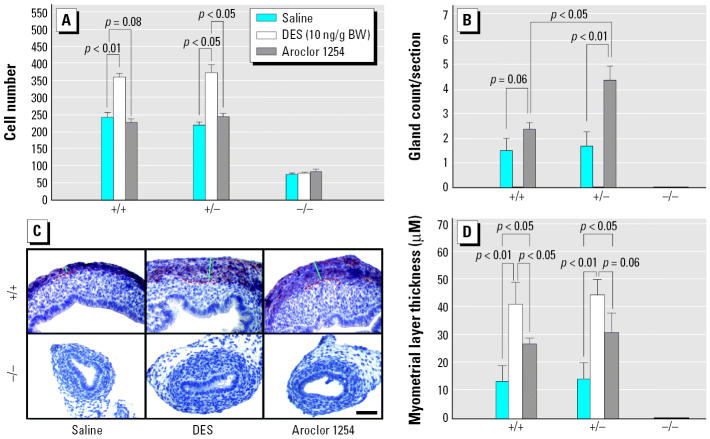
Uterine responses at PND6 to saline, DES, or and Aroclor 1254 exposure in wild-type, *Wnt7a* heterozygous, and mutant uteri involving changes in epithelial cell number (*A*), gland formation (*B*), and myometrial thickness (*C,D*). (*A*) Histogram showing changes in epithelial cell number. (*B*) Histogram showing changes in gland counts. DES exposure results in a complete block in gland formation in both *Wnt7a* wild-type and heterozygous uteri; in contrast, Aroclor 1254 exposure results in a significant increase in glandularity in wild-type and heterozygous uteri. In *Wnt7a* mutant uteri, no glands are formed under any conditions. (*C*) Immunohistochemistry for anti-smooth muscle actin revealing myometrial thickness in uterine samples. Both DES-exposed and Aroclor 1254–exposed uteri showed moderate to marked increases in myometrial thickness compared with saline-exposed controls. Bar = 100 μm. (*D*) Histogram showing changes in myometrial thickness measured from cross sections as shown in (*C*). Both low-level DES and Aroclor 1254 exposure resulted in an increase in myometrial thickness in both wild-type and heterozygous uteri, whereas the myometrium is undetectable in *Wnt7a* mutant uteri at this stage.

To precisely measure the morphologic responses to Aroclor 1254 and low levels of DES, we used morphometric analyses to determine the epithelial response (cell number and gland formation) as well as changes in the myometrium. As shown in Figure 3, DES induced an increase in epithelial cell number in both wild-type and *Wnt7a* heterozygous uteri at PND6, although as shown in Figure 2, the epithelial morphology was different between the two genotypes. In contrast, no change in epithelial cell number was seen between control and Aroclor 1254–exposed uteri of any genotype (Figure 3A). In control uteri, a few glands were beginning to form by PND6 (Figures 2A,3B); however, exposure to low levels of DES completely blocked gland formation (Figure 3B), consistent with our previous findings (Miller et al. 1998a). In contrast, exposure to Aroclor 1254 resulted in a significant increase in gland formation in both wild-type and *Wnt7a* +/− uteri, and this increase in gland formation was more pronounced in the *Wnt7a* heterozygote uteri (Figure 3B). The developing myometrium can be discerned by the general morphology of the outer cells of the uterus; however, to accurately measure myometrial thickness, we used immunohistochemistry for smooth muscle actin (Figure 3C). Exposure to either low levels of DES or Aroclor 1254 resulted in an increase in myometrial thickness, although the effects of DES were more pronounced compared with Aroclor 1254 (Figure 3D). Neither DES nor Aroclor 1254 had any effect upon the *Wnt7a* −/− uteri, which showed no overt myometrial formation under any conditions. Taken together, these data reveal that *Wnt7a* +/− uteri respond differently to DES or Aroclor 1254 compared with *Wnt7a* wild-type uteri, suggesting an increased sensitivity in the heterozygote state.

### Long-term effects of DES and Aroclor 1254 exposure on uterine morphology

**Figure 4:**
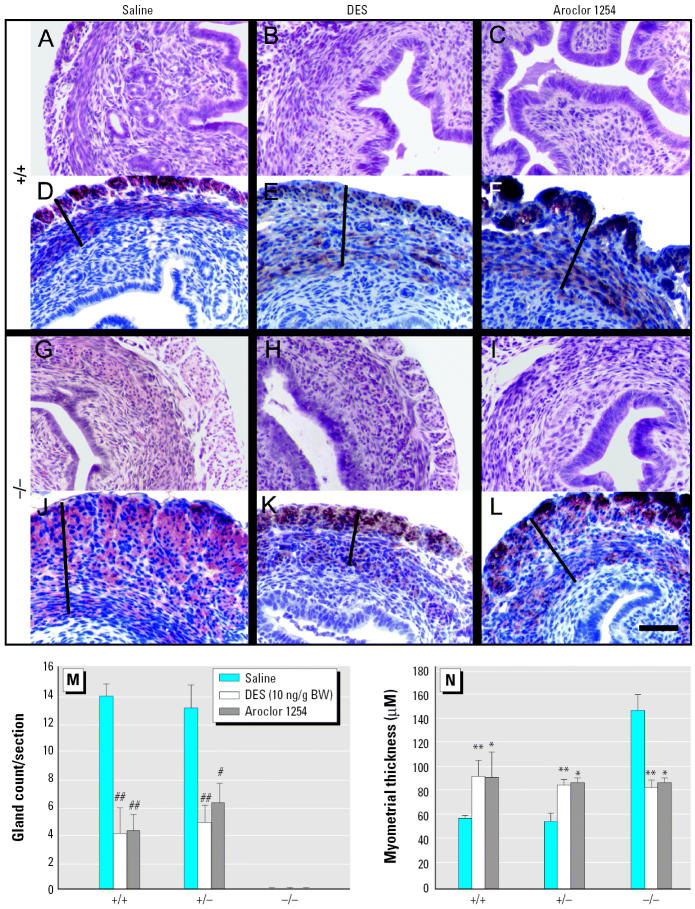
Changes in uterine myometrial thickness and glands in wild-type and *Wnt7a* mutant mice on PD30 after exposure to DES and Aroclor 1254 on PD1–PD5. DES and Aroclor 1254 exposure resulted in increased uterine myometrial thickness and a decrease in glands in wild-type mice (*A–F*), whereas the *Wnt7a* mutant mice (*G–L*) showed an increase in myometrial thickness (line, *D–F*, *J–L*) that occurred in the absence of exogenous treatment with DES or Aroclor 1254. The increase in myometrial thickness was blocked by DES or Aroclor 1254 exposure in the *Wnt7a* mutant. H&E-stained cross sections and immunohistochemistry for anti-smooth muscle actin are shown for wild-type (+/+; *A–C* and *D–F*) and *Wnt7a* mutant (−/−; *G–I* and *J–L*) uteri, respectively; bar = 100 μm. (*M*) Histogram depicting gland number obtained from cross sections similar to those shown in (*A–L*). (*N*) Myometrial thickness measured from cross sections similar to those shown in (*A–L*). *p < 0.05, **p < 0.01, ^#^*p* < 0.005, and ^##^*p* < 0.001 compared with saline.

Our results provide support that Aroclor 1254 exposure results in a down-regulation of *Wnt7a*, leading to phenotypic outcomes that strongly resemble those seen in response to DES. However, the morphologic effects of low level DES exposure and Aroclor 1254 were not identical. Specifically, both high- and low-level DES exposure blocked gland formation and had a more pronounced effect upon the myometrium and epithelial cell number at PND6 (Figures 2 and 3). Because gland formation and myometrial development initiate at the early postnatal stage we examined, we repeated our studies but examined uterine tissue on PND30, corresponding to a stage when cytodifferentiation is complete but tissues have not been subjected to high levels of endogenous circulating estrogens (Carta and Sassoon 2004). As shown in Figure 4, Aroclor 1254–exposed wild-type and *Wnt7a* +/− uteri that had increased numbers of uterine glands at PND6 show a striking decrease in gland number at PND30, mirroring the phenotype observed in the DES-exposed uteri. As previously reported, *Wnt7a* −/− uteri do not form uterine glands (Miller and Sassoon 1998; Miller et al. 1998a), and exposure to either DES or Aroclor 1254 does not change this phenotypic outcome. Exposure to DES or Aroclor 1254 increased myometrial thickness at PND30 in both *Wnt7a* wild-type (+/+) and +/− mice compared with saline controls (Figure 4). In the *Wnt7a* −/− mice, exposure to saline had no effect, and the myometrial layers were significantly larger, as previously reported (Carta and Sassoon 1994; Miller and Sassoon 1998). Surprisingly, although both DES and Aroclor 1254 had a pro-myogenic effect on *Wnt7a* +/+ and +/− uteri at PND30 compared with saline controls, *Wnt7a* −/−uteri exposed to either compound showed a decrease in the thickness of the myometrial layer compared with saline-exposed *Wnt7a* −/− uteri (Figure 4). The myometrial thickness of DES-exposed or Aroclor 1254–exposed *Wnt7a* −/− uteri, however, was similar to that of uteri from wild-type and heterozygote uteri similarly exposed at PND30 (Figure 4).

## Discussion

PCBs can act as endocrine-disrupting agents that presumably exert deleterious effects on the gonads and the reproductive tract because of their estrogenic and/or anti-estrogenic activity (Kholkute et al. 1994). The reproductive tracts in both males and females are particularly sensitive to hormonal disruption during perinatal development (Miller et al. 2004; Yang et al. 2005). Furthermore, reproductive tissues remain sensitive to circulating estrogenic compounds, which may pose an increased risk of reproductive tract cancers during adult life (Hatch et al. 2000; Shekhar et al. 1997a). Despite several decades of studies focused upon the developmental impact of PCB exposure upon the female reproductive tract, the molecular and cellular pathways that lead to reproductive tract malformations, dysfunctions, and cancer progression after PCB exposure are poorly understood. In contrast to the situation with PCBs and other “weak” estrogenic compounds, a strong mechanistic and epidemiologic link exists between exposure to the synthetic estrogen DES during perinatal development and malformations of the uterus and the subsequent appearance of cancers in the adult (Sassoon 1999). Work from our laboratory has previously implicated the *WNT* gene pathway as a critical target that is disrupted by perinatal DES exposure in the female reproductive tract (Carta and Sassoon 2004; Kitajewski and Sassoon 2000; Miller and Sassoon 1998; Miller et al. 1998a).

*WNT* genes direct the proper cyto-differentiation and overall development of the murine female reproductive tract, which is relatively undifferentiated at birth and is subject to specific epithelial–mesenchymal interactions and to regulation by sex-steroid hormones during development and adult life. Postnatal reproductive tract development proceeds in the absence of high levels of endogenous circulating estrogens and is disrupted by exposure to estrogenic chemicals during this period. By employing mouse genetic models, we and other groups have demonstrated that *Wnt7a* is a central target gene in the DES response after perinatal exposure and that the deregulation of *Wnt7a* is dependent upon the presence of the estrogen receptor α-isoform (Couse and Korach 2004; Miller et al. 1998a; Sassoon 1999). Thus, *Wnt7a* down-regulation is a key response to estrogen in the uterus. The central role of *Wnt7a* is underscored by the observation that perinatal DES exposure down-regulates *Wnt7a*, leading to a female reproductive tract phenotype that closely resembles that observed in *Wnt7a* nullizygous mice (Miller et al. 1998a; Sassoon 1999). Additionally, exposure of *Wnt7a* mutant uteri to estrogens fails to elicit a uterotrophic response (Carta and Sassoon 2004). Because *Wnt7a* down-regulation is an immediate molecular response to estrogen via estrogen-receptor–dependent pathways, we tested whether *Wnt7a* down-regulation could be used as a bioassay to better address the mechanism of action of suspected weak estrogenic compounds or mixtures such as PCBs. In this study, we demonstrated that Aroclor 1254 (PCBs commercial mixture) exposure results in a down-regulation of *Wnt7a*.

Our previous studies were performed using high doses of DES, reflecting those administered to pregnant women; however, for this study we chose to compare PCB exposure with low-level DES exposure to ascertain if lower levels of DES result in *Wnt7a* down-regulation. We found that Aroclor 1254 and low-level DES exposure resulted in down-regulation of *Wnt7a* and in the subsequent induction of overlapping phenotypic malformations in the uterus, including reduced gland formation and an increase in thickness of the smooth muscle layer, by PND30 compared with saline-treated mice. Finally, we found that neither DES nor Aroclor 1254 exposure altered the morphology of uteri from *Wnt7a* mutant mice, although we observed that these mice already displayed reduced gland formation and a disorganized myometrium due to the absence of *Wnt7a* expression, as previously reported (Miller and Sassoon 1998; Miller et al. 1998a; Sassoon 1999). Taken together, our data demonstrate that Aroclor 1254 and low-level DES exposure affect the perinatal uterus via the *Wnt7a* signaling pathway. The effects of low-level DES exposure and Aroclor 1254 exposure were more pronounced in *Wnt7a* +/− reproductive tracts, including pronounced luminal epithelium stratification. These observations confirm that deregulation of the *Wnt7a* pathway is a central response to PCB exposure in the developing reproductive tract. In addition, the observed increase in sensitivity of the *Wnt7a* heterozygous reproductive tract supports the model that genetic predisposition plays a key role in the response to environmental endocrine disruptors. Given that *WNT* signaling involves a large variety of gene products, there is likely to be a complex genetic background effect in human populations in response to environmental endocrine disruptors.

Assessment of the potential dangers and estrogenic risks of environmental contaminants has heavily relied on rodent models in which neonatal mice are exposed and then end points are measured, including the uterotrophic response and morphologic abnormalities within the reproductive tract (Gray et al. 2004; Spearow and Barkley 2001). These studies involve time-consuming histologic analyses and large numbers of animals. As demonstrated in our studies, the morphologic effects of PCBs are not as severe as those seen in response to strong estrogenic stimuli, such as high or moderate levels of DES, and are therefore difficult to assess. The studies presented here demonstrate that a rapid and simple analysis of *Wnt7a* message levels in the reproductive tract after chemical exposure could be a reliable bioassay to measure potential endocrine disruptors. The down-regulation of *Wnt7a* expression in the uterine luminal epithelium was observed in response to both Aroclor 1254 and DES exposure; however, the initial morphologic responses seen at PND6 were different. Specifically, DES exposure induced significant epithelium stratification, whereas we found no major changes in the luminal epithelium in PCB-exposed uteri, with the notable exception that there is a significant increase in uterine gland number in both wild-type and *Wnt7a* heterozygous mice. The induction of specific outcomes of PCBs, such as a transient increase in gladularity, may reflect the fact that the PCB mixture used in this study, namely, Aroclor 1254, is a complex mixture of > 40 congeners that may induce a variety of effects that are not evoked by estrogen. These observations suggest that PCB exposure activates both *Wnt7a*-dependent and -independent signaling pathways. However, the observation that neonatal exposure to both Aroclor 1254 and DES results in a similar phenotype by PND30 suggests that the transient down-regulation of *Wnt7a* triggers a permanent reprogramming of the developing reproductive tract. Work by us and others indicates that this reprogramming culminates in the development of highly abnormal tissue with spontaneous neoplasias of variable frequencies. The future assessment of endocrine disruptors and their potential risks will depend upon a thorough mechanistic understanding of the molecular and cellular processes that result from the deregulation of normal cell signaling events guiding reproductive tract development.
